# Exploring influential factors in the self-assessment of life satisfaction among Chinese elderly: a structural equation modeling analysis

**DOI:** 10.3389/fpsyt.2024.1349346

**Published:** 2024-04-19

**Authors:** Jun Yan, Suzhen Wang, Chang Liu, Yuanan Lu

**Affiliations:** ^1^ Institute of Traditional Chinese Medicine and Health Development, Jiangxi University of Chinese Medicine, Nanchang, China; ^2^ Institute of Chinese Medical Sciences, University of Macau, Macao, Macao SAR, China; ^3^ Office of public health Studies, University of Hawaii at Manoa, Honolulu, HI, United States

**Keywords:** Chinese elderly, self-assessed life satisfaction (SALS), region, structural equation modeling(SEM), health promotion

## Abstract

The aging problem is becoming more and more prominent globally. Attention to the quality of life and related health improvement among the elderly has become an important issue in modern society. This study utilized a tracking survey conducted in 2017-2018, involving 9,327 Chinese older adults, to examine health influencing factors, and applied structural equation modeling to analyze the influencing factors on the self-assessment of life satisfaction among older adults in different regions (cities, counties, and villages) in China. This study revealed that economic status, psychological status, personal situation, life behaviors, and child care are important influences on older people’s self- assessed life satisfaction. There is a positive correlation between economic status, psychological status, child care and the results of the self-assessment of life satisfaction of the elderly. Psychological status and child care have a greater impact on the self-assessment of life satisfaction among the elderly in urban areas compared to villages and towns. The influence of economic status on the self-assessment of life satisfaction of the elderly is lower in urban areas than in rural areas. There is a significant difference in the influence of personal situations on the self-assessment of life satisfaction among the elderly. Additionally, older individuals tend to report higher levels of self-assessment of life satisfaction. Furthermore, female elderly individuals tend to report higher levels of satisfaction compared to males. Findings from this study indicate that improving health self-assessment in older adults requires targeted efforts based on different geographic areas of life and the age stages of older adults, and more attention needs to be paid to men who are just entering old age.

## 1 Introduction

The self-assessment of life satisfaction among older adults serves as a crucial indicator of their overall health. The unprecedented aging of the global population, particularly in developing nations, has garnered significant attention. According to the United Nations (2015), the elderly population is projected to surge from 901 million to 1.4 billion by 2030 and is expected to reach approximately 2 billion by 2050, constituting 20% of the world’s population (1). Statistics from China’s National Bureau of Statistics (NBS) reveal that, in 2022, the population aged 65 and above will stand at 210 million, accounting for 14.87% of China’s total population. The General Report of the National Office for the Elderly on the Study of National Strategies for Coping with Population Aging, released in 2018, forecasts three peaks in the growth of China’s elderly population over the next 40 years, with fluctuations exceeding 50% in both number and proportion, accelerating the nation’s overall rate of population aging (2). Highlighting this demographic shift, the World Health Organization (WHO) reported that the growth of healthy life expectancy among the Chinese population has accelerated in recent years. China has entered an aging society and is anticipated to experience rapid aging in the future (3). Consequently, the focus of contemporary society has shifted towards promoting the health, as well as enhancing the quality of life, for the elderly. The concern for the well-being of older adults and the ongoing efforts to improve their health have become central themes in today’s societal discourse.

Self-reported life satisfaction and self-reported health in older adults can independently predict disease incidence and mortality in older adults (4). Strengthening the study of life satisfaction of older adults is of positive significance for the daily health management of older adults. Evaluation of life satisfaction of older adults is a comprehensive assessment, which is influenced by a variety of factors including age, education, and gender (5). Research on life satisfaction of older adults has been carried out earlier in the United States, Switzerland, Canada, Germany, Japan and other regions with the main focus on the connotation of self-assessment of life satisfaction of older adults, evaluation activities, and factors affecting the self-assessment of older adults’ health (6–8). In recent years, scholars have explored the influence of self-assessment of life satisfaction on older adults focusing on psychological, behavioral, demographic, social support, health care, and many other aspects (9–11). Through empirical studies, researchers have further demonstrated the important role of the above influences on the improvement of health of the coming generations (12–16). In this study, the self-assessment survey on life satisfaction of 9,327 elderly people in China in 2018 was used as the research object, and the structural equation modeling method was applied to study the influence of related factors on the self-assessment of life satisfaction of elderly people. And we further constructed three-level structural equation modeling at the city, town, and village levels to explore the differences in the self-assessment of life satisfaction of the elderly in different administrative regions, and to more fully validate the research results.

## 2 Method

### 2.1 Subjects

The data for this study were sourced from the China Senior Health Survey (CLHLS), which comprehensively covered 23 provinces, municipalities, and autonomous regions. The survey targeted individuals aged 60 years and above, with those unable to independently respond to the questionnaire receiving assistance from their adult children. The survey encompassed a diverse range of topics, including self-assessed health status, personality psychology, behavior, and lifestyle, living environment, and economic status. Scientifically designed, systematic, and representative, the survey offers valuable insights into the health status of the elderly population in China. Initiated in 1998 as a baseline study with 9093 participants, the CLHLS conducted six subsequent follow-up surveys spanning from 2000 to 2018 (17). Each follow-up survey incorporated additional samples based on evolving survey needs. The 2017-2018 follow-up survey, specifically utilized in this study, included 12,411 new samples. This research exclusively employed the latest tracking survey data. Adhering to the study’s inclusion criteria, missing or invalid sample data for key indicators such as quality of life assessment, depression, education, and area of residence were meticulously excluded. Ultimately, 9327 subjects were retained for the analysis.

### 2.2 Method

Structural Equation Modeling (SEM) was employed to scrutinize the influencing factors of Self-Assessment of Life Satisfaction (SALS) among the elderly. SEM, a multivariate statistical model originally developed in the early 1970s for fit modeling in social science (18), proves particularly useful for examining variables that cannot be directly measured or observed. Encompassing regression analysis, factor analysis, path analysis, and latent growth curve modeling, SEM facilitates parameter estimation of systems of joint equations. Widely utilized for observing functions of multiple variables and exploring multivariate associations (19, 20), SEM emerges as a comprehensive tool in understanding the complexities of SALS influencing factors among the elderly.

### 2.3 Influencing factors

The growing elderly population poses a great challenge to social development, and the increasing base of the elderly population and strained healthcare resources are affecting the quality of life of the elderly (21). Research on life satisfaction among older adults is increasing, and influences such as age (22), gender (23), psychological condition (24), economic status (25), health-related behaviors (26), and child care (27) are often included as key influences in studies, depending on the topic of study. Relevant studies have also demonstrated the relevance of the above influences on the self-assessment of life satisfaction in older adults. (28–30). Based on this, combined with the objectives of this study and the question design of the Chinese Longitudinal Healthy Longevity Survey (CLHLS) in the relevant dimensions, we summarized the influencing factors of self-assessment of life satisfaction of the elderly into five dimensions: personal status (PS), economic situation (ES), living behavior (LB), mental state (MS), and child care (CC). Among these factors, personal status encompasses gender (I1), age (I2), education (I3), and place of residence (I4); economic situation includes the sufficiency of family economic income (E1) and self-assessment of the family’s economic level at the local level (E2); living behavior covers daily exercise (B1), recreational activities (B2), alcohol consumption (B3), and smoking (B4); recreational activities specifically involve reading books and newspapers, keeping pets, and socializing; mental state comprises depression (M1) and anxiety (M2); and child care includes home visits (C1), telephonic contact (C2), and annual financial assistance (C3).

### 2.4 Quality control

In this study, several control approaches were implemented to ensure the quality and accuracy of our findings and conclusions. 1) Two-person screening and cross-comparison were employed to enhance the accuracy of data screening. 2) According to the Structural Equation Model (SEM), the fitness index criteria were set as follows: Standardized Root Mean Square Residual (SRMR) required to be less than 0.08; Goodness of Fit Index (GFI), Adjusted Goodness of Fit Index (AGFI), Normed Fit Index (NFI), Incremental Fit Index (IFI), and Comparative Fit Index (CFI) all needed to exceed 0.9. Additionally, the Parsimony Goodness of Fit Index (PGFI) was expected to be above 0.5. 3) Various fitting indexes were utilized to assess the suitability of the constructed SEM for correlation analysis between Subjective Well-being (SRH) and its related influencing factors, as outlined by Stein et al. (18).

### 2.5 Statistical analysis

This study utilized SPSS 22.0 software for general descriptive analysis. AMOS 20.0 statistical software was employed to construct and evaluate the structural equation model (SEM), including fitting assessment. The Adjusted Model equations were optimized through adjustments to data adaptation or causal paths, including the deletion of insignificant causal paths. A significance level of *P* < 0.05 was considered for determining statistical differences.

## 3 Results

### 3.1 General information of participants

A total of 9,327 participants were included in this study. Demographic information for all sample participants was meticulously categorized and tabulated based on personal circumstances, economic status, lifestyle choices, psychological well-being, and childcare responsibilities. Among the 9,327 enrolled subjects, 4,293 (46.03%) were male, and 5,034 (53.97%) were female. Age distribution revealed that 2,504 (26.85%) fell between 65 and 74 years, 2,757 (29.56%) between 75 and 84 years, 2,314 (24.81%) between 85 and 94 years, and 1,752 (18.78%) were aged 95 and above. Participants self-assessed their quality of life, with 6,624 individuals (71.02%) reporting a positive evaluation, and 2,703 individuals (28.98%) indicating a less favorable assessment (see **Table 1**).

**Table 1:** General information of enrolled subjects (n=9237).

Variable	Case (n)	%	Varieable	Case (n)	%
**Personal situation**			**economic situation**		
**Sex**			**Economic Source**		
Male	4293	46.03	Enough	8122	87.08
Female	5034	53.97	Not enough	1205	12.92
**Age**			**Economic level** ^a^		
65~74	2504	26.85	Wealthier	1919	20.57
75~84	2757	29.56	General	6525	69.96
85~94	2314	24.81	Poorer	883	9.47
≥95	1752	18.78	**Mental state (feeling)**		
** Living area**			**Depression**		
City	2305	24.71	Rarely	6830	73.23
Town	3061	32.82	Sometimes	2362	25.32
Village	3961	42.47	Often	135	1.45
**Education**			** anxiety**		
No	4094	43.92	Rarely	8216	88.09
Yes	5231	56.10	Sometimes	923	9.90
**Health-related behavior**			Often	188	2.01
**Smoking**			**Child Care**		
Yes	1506	16.15	**home visits**		
No	7821	83.85	Often	8584	92.03
**Liqueur**			Rarely	743	7.97
Yes	1447	15.51	**telephonic contact**		
No	7880	84.49	Often	8630	92.53
**Exercise**			Rarely	697	7.47
Yes	3250	34.85	**financial giving per year (yuan)** ^b^		
No	6077	65.15	<1000	1534	16.45
**Entertainment**			1000~5000	3398	36.43
Yes	8376	89.80	>5000	4395	47.12
No	951	10.19			

a As compared with the local level.

b 1,000 yuan equals about $137.

### 3.2 The establishment and refinement of the model

#### 3.2.1 Model establishment

The initial model was formulated grounded in the existing research framework. Latent variables influencing the model encompass personal status, economic status, lifestyle choices, mental well-being, and childcare, with the ultimate variable being the self-assessed life satisfaction of elderly individuals. The original questionnaire delineated self-assessment of life satisfaction into five dimensions: excellent, good, average, not so good, and poor. Considering the limited sample size under certain dimensions, we consolidated some categories; specifically, the original dimensions of excellent and good were amalgamated into self-assessed good, while average, not so good, and poor were merged into self-assessed poor.

To investigate the variations in self-assessed life satisfaction groups among elderly individuals in different regions, we established three structural equation models based on the residential divisions of the survey samples, namely cities, towns, and villages, designated as Model I, II, and III, respectively. Concurrently, to delve deeper into and validate regional disparities in the influencing factors of elderly individuals’ self-assessed life satisfaction, we developed a nationwide structural equation model using the overall dataset, denoted hereafter as Model IV.

#### 3.2.2 Model fitness assessment

To evaluate the overall fitness of the model, a test was conducted using the initially established model in AMOS 20.0 statistical software. Criteria for a good fit included SRMR<0.08, GFI, AGFI, NFI, RFI, IFI, and CFI>0.9, and PGFI>0.5. Taking model I as an illustration, the results indicated that certain initial model fitting index parameters did not meet the specified criteria. For instance, SRMR was 0.054, exceeding the threshold of 0.05, and NFI, IFI, and CFI were 0.782, 0.720, and 0.796, respectively, all falling below 0.9. Consequently, model correction was deemed necessary to achieve an optimal fit.

#### 3.2.3 Model modification

**Figure 1:**
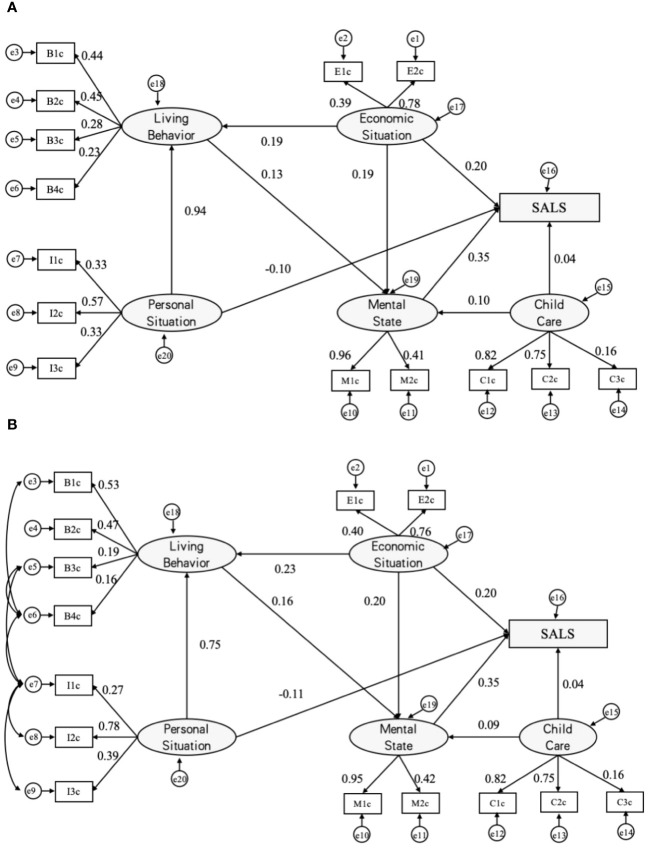
Analysis diagrams of SALS paths (MODEL I). Initial paths **(A)** and modified SALS paths **(B)**. a β’ value is used for each path and e1~e20 are error variables; SALS = Self-assessed life satisfaction; B1=exercising, B2=recreational activities, B3=drinking, and B4=smoking; E1=income, E2=economic status; I1=education, I2=gender, and I3=age; M1=depression, M2=anxiety,; C1=home visits, C2=telephonic contact and C3=financial giving.

**Figure 2:**
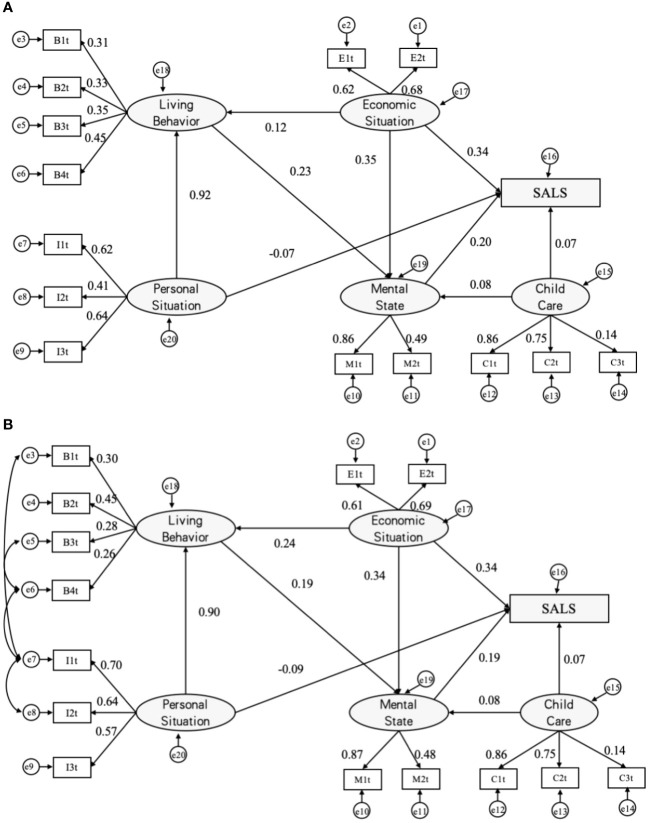
Analysis diagrams of SALS paths (MODEL II). Initial paths **(A)** and modified SALS paths **(B)**. a β’ value is used for each path and e1~e20 are error variables; SALS = Self-assessed life satisfaction; B1=exercising, B2=recreational activities, B3=drinking, and B4=smoking; E1=income, E2=economic status; I1=education, I2=gender, and I3=age; M1=depression, M2=anxiety,; C1=home visits, C2=telephonic contact and C3=financial giving.

**Figure 3:**
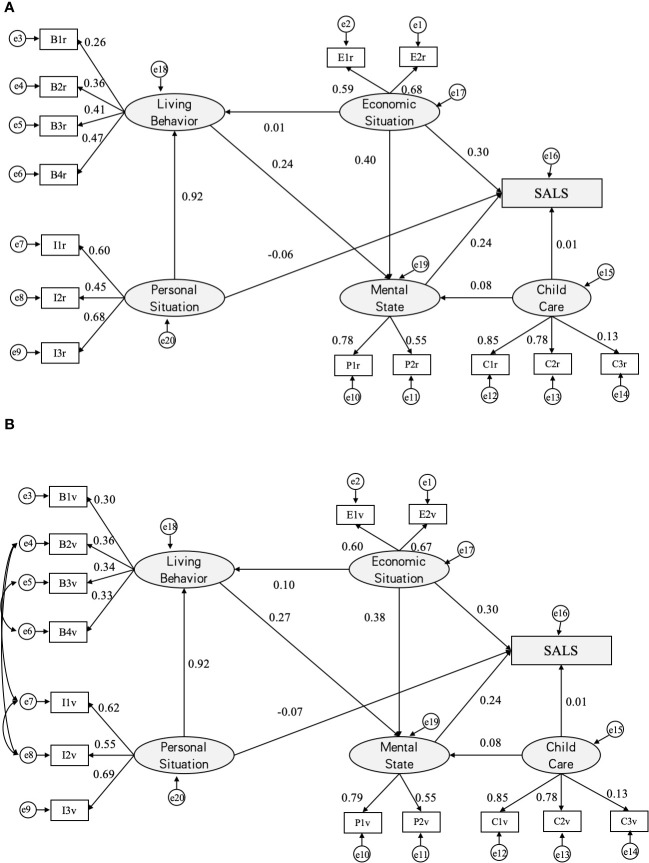
Analysis diagrams of SALS paths (MODEL III). Initial paths **(A)** and modified SALS paths **(B)**. a β’ value is used for each path and e1~e20 are error variables; SALS = Self-assessed life satisfaction; B1=exercising, B2=recreational activities, B3=drinking, and B4=smoking; E1=income, E2=economic status; I1=education, I2=gender, and I3=age; M1=depression, M2=anxiety,; C1=home visits, C2=telephonic contact and C3=financial giving.

**Figure 4:**
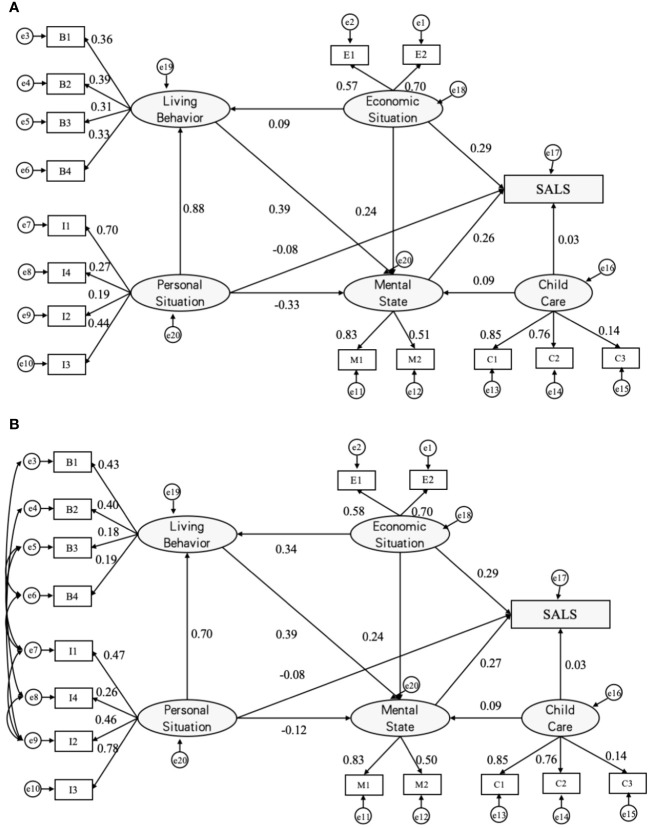
Analysis diagrams of SALS paths (MODEL IV). Initial paths **(A)** and modified SALS paths **(B)**. a β’ value is used for each path and e1~e20 are error variables; SALS = Self-assessed life satisfaction; B1=exercising, B2=recreational activities, B3=drinking, and B4=smoking; E1=income, E2=economic status; I1=education, I2=gender, I3=age and I4=place of residence; M1=depression, M2=anxiety,; C1=home visits, C2=telephonic contact and C3=financial giving.

Based on the initial test results, adjustments were made to rectify the model’s paths. For model 1, covariance links were introduced between e6 and e3, e5, and e7, and between e7 and e8 and e9. The revised model path map was generated (see **Figure 1B**). Subsequent fit testing revealed significant improvements, with SRMR reduced to 0.037 (<0.05), and GFI, AGFI, NFI, IFI, and CFI surpassing 0.9. PGFI was 0.621 (>0.5). All indicators met the stipulated standards, indicating the overall satisfactory fitting of the structural equation model (see **Tables 2**, **3**; **Figures 1**–**4**).

**Table 2:** Index of structural fitness test.

Inspection index	SRMR	GFI	AGFI	PGFI	NFI	IFI	CFI
**Initial equation (City)**	0.054	0.949	0.926	0.649	0.782	0.797	0.796
**Adjusted equation (City)**	0.037	0.980	0.969	0.621	0.916	0.933	0.932
**Initial equation (Town)**	0.049	0.953	0.931	0.651	0.843	0.853	0.812
**Adjusted equation (Town)**	0.037	0.981	0.970	0.638	0.933	0.944	0.944
**Initial equation (village)**	0.050	0.952	0.930	0.651	0.844	0.801	0.853
**Adjusted equation (village)**	0.038	0.980	0.969	0.637	0.932	0.909	0.941
**Initial equation (Nationwide)**	0.056	0.944	0.920	0.666	0.797	0.801	0.801
**Adjusted equation (Nationwide)**	0.039	0.982	0.973	0.643	0.932	0.936	0.936
**Fitness requirement**	<0.05	>0.9	>0.9	>0.5	>0.9	>0.9	>0.9

SRMR, Standardized Root Mean Square Residual; GFI, Goodness of Fit Index; AGFI, Adjusted Goodness of Fit Index; PGFI, Parsimony Goodness of Fit Index; NFI, Normed Fit Index; IFI, Incremental Fit Index; CFI, Comparative Fit Index.

**Table 3:** Index of internal structural fitness of structural equation.

Latent V	City						Town					
	Observed V	USCE	S.E.	C.R.	*P*	SCE	Observed V	USCE	S.E.	C.R.	*P*	SCE
**Personal Status**	I3c	1.000				0.782	I3t	1.000				0.636
	I2c	0.192	0.019	9.888	<001	0.390	I2t	0.419	0.025	16.507	<001	0.567
	I1c	0.162	0.026	6.196	<001	0.274	I1t	0.512	0.028	18.042	<001	0.701
	–	–	–	–	–	–	–	–	–	–	–	–
**Economic Situation**	E1c	1.000				0.398	E1t	1.000				0.609
	E2c	4.252	0.626	6.795	<001	0.763	E2t	1.695	0.104	16.243	<001	0.689
**Living Behavior**	B4c	1.000				0.164	B4t	1.000				0.256
	B3c	1.266	0.253	4.995	<001	0.188	B3t	1.048	0.111	9.473	<001	0.279
	B2c	2.461	0.465	5.294	<001	0.468	B2t	1.378	0.144	9.582	<001	0.453
	B1c	5.355	1.018	5.262	<001	0.533	B1t	1.429	0.161	8.900	<001	0.303
**Mental State**	M1c	1.000				0.950	P1t	1.000				0.873
	M2c	0.375	0.038	9.370	<001	0.418	P2t	0.568	0.047	12.192	<001	0.482
**Child Care**	C1c	1.000				0.823	C1t	1.000				0.856
	C2c	0.829	0.103	8.047	<001	0.746	C2t	0.870	0.096	9.049	<001	0.751
	C3c	0.603	0.099	6.061	<001	0.161	C3t	0.444	0.071	6.265	<001	0.142
Latent V	Village						Nationwide					
	Observed V	USCE	S.E.	C.R.	*P*	SCE	Observed V	USCE	S.E.	C.R.	*P*	SCE
**Personal Status**	I3v	1.000				0.549	I2	1.000				0.461
	I2v	0.588	0.033	17.580	<001	0.687	I3	0.781	0.033	23.349	<0.01	0.777
	I1v	0.526	0.026	20.028	<001	0.618	I1	0.475	0.018	26.343	<0.01	0.470
	–	–	–	–	–	–	I4	0.417	0.024	17.154	<0.01	0.257
**Economic Situation**	E1v	1.000				0.595	E1	1.000				0.575
	E2v	1.668	0.099	16.790	<001	0.674	E2	1.934	0.083	23.221	<0.01	0.695
**Living Behavior**	B4v	1.000				0.329	B4	1.000				0.190
	B3v	0.997	0.083	11.997	<001	0.338	B3	0.941	0.088	10.645	<0.01	0.182
	B2v	0.928	0.086	10.730	<001	0.357	B2	1.741	0.148	11.745	<001	0.428
	B1v	1.081	0.108	10.050	<001	0.301	B1	2.924	0.248	11.807	<0.01	0.403
**Mental State**	P1v	1.000				0.785	P1	1.000				0.830
	P2v	0.660	0.039	16.927	<001	0.548	P2	0.573	0.025	22.987	<0.01	0.502
**Child Care**	C1v	1.000				0.850	C1	1.000				0.846
	C2v	0.900	0.096	9.369	<001	0.777	C2	0.872	0.057	15.180	<0.01	0.761
	C3v	0.399	0.059	6.775	<001	0.134	C3	0.458	0.042	10.967	<0.01	0.143

Latent V, latent variable; Observed V, observed variable; USCE, unstandardized coefficient; SCE, Standardized coefficient; S.E., standard error; C.R., construct reliability.

### 3.3 Analysis of factors influencing elderly self-assessment of life satisfaction

#### 3.3.1 Elderly residents in urban

Model I reveal a significant correlation among latent variables representing mental state, economic situation, and personal situation concerning outcome variables for urban elderly individuals. The influence of each factor varies, with the descending order of impact as follows: mental state (*β’* = 0.35), economic situation (*β’* = 0.20), and personal situation (*β’* = -0.11). Additionally, life behavior indirectly affects the outcome latent variable through the mediation of mental state. Notably, the correlation between the latent variable for child care and the outcome latent variable is not statistically significant (*P* > 0.05), as detailed in **Table 4**. This suggests that the mental state significantly impacts the elderly’s perception of health, exhibiting a positive correlation. Specifically, a better mental state is associated with a higher self-assessment of life satisfaction.

**Table 4:** Indexes of fitness between latent variables of structural equations.

			City					Town				
			USCE^a^	S.E.	C.R.	*P*	SCE^b^	USCE^a^	S.E.	C.R.	*P*	SCE^b^
PS	->	LB	0.044	0.009	4.954	<0.01	0.746	0.133	0.019	5.720	<0.01	0.900
	->	SALS	-0.059	0.013	-4.531	<0.01	-0.113	-0.058	0.013	-4.477	<0.01	-0.086
ES	->	MS	0.943	0.150	6.294	<0.01	0.196	0.657	0.057	11.519	<0.01	0.338
	->	LB	0.123	0.030	4.112	<0.01	0.230	0.109	0.019	5.720	<0.01	0.236
	->	SALS	0.949	0.139	6.848	<0.01	0.201	0.723	0.060	12.087	<0.01	0.343
LB	->	MS	1.401	0.364	3.846	<0.01	0.155	0.792	0.120	6.594	<0.01	0.189
MS	->	SALS	0.344	0.042	8.261	<0.01	0.352	0.210	0.031	6.860	<0.01	0.193
CC	->	MS	0.192	0.052	3.683	<0.01	0.093	0.146	0.041	3.568	<0.01	0.081
	->	SALS	0.077	0.045	1.731	0.084	0.038	0.130	0.038	3.409	<0.01	0.066
			Village					Nationwide				
			USCE^a^	S.E.	C.R.	P	SCE^b^	USCE^a^	S.E.	C.R.	P	SCE^b^
PS	->	LB	0.169	0.014	12.157	<0.01	0.792	0.099	0.008	11.896	<0.01	0.702
	->	SALS	-0.053	0.015	-3.635	<0.01	-0.068	-0.098	0.030	-3.222	<0.01	-0.122
ES	->	MS	0.670	0.051	13.099	<0.01	0.381	-0.072	0.011	-6.581	<0.01	-0.079
	->	LB	0.056	0.019	2.953	<0.01	0.097	0.700	0.036	19.605	<0.01	0.340
	->	SALS	0.641	0.055	11.719	<0.01	0.303	0.675	0.037	18.243	<0.01	0.288
LB	->	MS	0.816	0.097	8.445	<0.01	0.267	2.224	0.308	7.229	<0.01	0.392
MS	->	SALS	0.295	0.032	9.216	<0.01	0.244	0.303	0.020	15.524	<0.01	0.265
CC	->	MS	0.135	0.035	3.891	<0.01	0.083	0.151	0.024	6.397	<0.01	0.087
	->	SALS	0.021	0.033	0.632	0.528	0.010	0.068	0.022	3.163	<0.01	0.035

a USCE, unstandardized coefficient.

b SCE, Standardized coefficient.

PS, personal status; ES, economic situation; LB, living behavior; MS, mental state; CC, child care.

Furthermore, a positive correlation is observed between economic status, life behaviors, and the self-assessment of life satisfaction among the elderly. This implies that improved economic status and the adoption of health-related behaviors contribute to a higher self-assessment of life satisfaction. Conversely, personal situation exhibits a negative correlation with the self-assessment of life satisfaction among the elderly, indicating that life satisfaction tends to increase as individuals age.

**Table 4** illuminates the impact of observed variables within each latent influential factor on its corresponding latent variable. Specifically, within the personal situation category, the path coefficient from the observed variable of age to the latent variable of personal situation is 0.78. This surpasses the path coefficients of education and gender to personal situation, underscoring that age exerts the most significant influence on personal situation. Within the economic situation indicators, the highest path coefficient (0.76) is attributed to the self-assessment of the economic level of the elderly’s family at the local level, signifying that superior economic circumstances contribute significantly to enhancing the self-assessment of the elderly’s life satisfaction. Simultaneously, the path coefficient of the indicator gauging personal income’s ability to meet the elderly’s life needs is 0.40, suggesting a positive correlation between income sufficiency and self-assessment of life satisfaction.

Moreover, under the life behavior indicator, daily exercise and recreational activities exhibit path coefficients of 0.47 and 0.53, respectively, ranking as the top two influencers. This implies that engaging in exercise and recreational activities holds greater sway in improving the self-assessment of life satisfaction among the elderly compared to the influence of smoking and drinking habits.

In the realm of mental state, the observed variable of depression demonstrates a substantial path coefficient of 0.95 on the latent variable of psychological state. This underscores that the mental well-being of the elderly is significantly impacted by depression, suggesting that depression plays a pivotal role in shaping the self-assessment of life satisfaction among the elderly.

Lastly, within the children’s care indicator, visiting and communication emerge as the top two influential observed variables in the latent variable, boasting path coefficients of 0.82 and 0.75, respectively. These figures significantly surpass the path coefficient of 0.16 for children’s financial support, indicating that visiting and communication wield a more profound influence on the elderly’s self-assessment of life satisfaction than financial support from their children.

#### 3.3.2 Elderly residents in townships and villages

Model II reveals significant correlations between the latent variables of mental status, economic situation, lifestyle choices, and childcare responsibilities among elderly individuals in villages and townships, and the outcome variables. The strength of influence, ranked from highest to lowest, was observed in the following order: economic status (*β’*=0.34), mental status (β’=0.19), personal situation (*β’*=-0.09), and child care (*β’*=0.07). Notably, the life behaviors of the elderly in townships impacted the self-assessment of life satisfaction through psychological states, as depicted in **Table 4**.

In townships, economic status emerged as the most influential factor in the self-assessment of life satisfaction among the elderly. This suggests that the local-level self-assessment of the economic situation in elderly families in townships, along with the sufficiency of elderly income, holds a comparable degree of influence on their life satisfaction self-assessment. Concerning lifestyle choices, the path coefficient for daily recreational activities was 0.45, indicating a relatively greater impact compared to the path coefficients of physical exercise, smoking, and drinking on latent variables. This underscores that engaging in daily recreational activities among elderly individuals in townships is more likely to enhance their self-assessment of life satisfaction.

Examining the influence of psychological states, personal situations, and latent variables of childcare on the self-assessment of life satisfaction among the elderly in townships, the impact mirrors that observed in cities. Similarly, in villages, psychological states, economic status, lifestyle choices, and childcare latent variables exhibit significant correlations with the outcome variables. The hierarchical roles of each influencing factor parallel those observed in townships. However, in terms of lifestyle behaviors among village elderly, the path coefficients for daily recreation, physical exercise, smoking, and drinking are comparable, with values of 0.36, 0.30, 0.33, and 0.34, respectively. This suggests that these behaviors exert similar levels of influence on the life satisfaction of elderly individuals in villages.

Model III shows that there is a significant association between each of the factors related to rural elderly on the self-assessment of life satisfaction of the elderly. The factors from strongest to weakest were economic status (β’=0.30), psychological status (β’=0.24), personal situation (β’=-0.07), and child care (β’=0.01). The correlation results were similar to those of the town elderly.

#### 3.3.3 Elderly in the country

Findings from Models I, II, and III indicate that the influencing factors impacting the self-assessment of life satisfaction vary among elderly groups in different regions. To further validate the influence of these common factors on the self-assessment of life satisfaction across diverse regions, we incorporated the place of residence of elderly groups as an observational variable within the latent variable of personal situation. Subsequently, we constructed Model IV, a comprehensive structural equation model based on the nationwide elderly group.

The outcomes presented in **Table 3** elucidate the self-assessed influences on life satisfaction within the national elderly group. In descending order of influence, these factors include economic status (*β’* = 0.29), mental status (*β’* = 0.27), personal situation (*β’* = -0.08), and childcare (*β’* = 0.03). Notably, there exists a significant correlation between each latent variable and the outcome variable. Within the economic status latent variable, the path coefficient related to the self-assessment of the family’s economic level at the local level stands notably high at 0.70, underscoring the substantial impact of this observational variable on the elderly’s self-assessment of life satisfaction. In the realm of mental status, the elderly’s depression situation emerges as a prominent influencing factor, with a substantial path coefficient of 0.83.

Regarding the personal situation latent variable, the addition of the place of residence of the elderly group as an observational variable reveals a negative correlation with mental status. Further analysis, illustrated in **Figure 3**, demonstrates that the level of depression, as indicated by the observational variable, diminishes gradually with the city and town residence areas.

## 4 Discussion

### 4.1 Key factors influencing elderly life satisfaction

#### 4.1.1 Economic status and its positive relationship with elderly life satisfaction

Economic status demonstrates a positive correlation with the self-assessment of life satisfaction among the elderly. Older individuals whose income meets daily needs and have a favorable economic self-assessment tend to report higher life satisfaction (31). With increasing societal attention to aging issues, the growing economic support for the elderly plays a crucial role. Numerous studies consistently emphasize the significant impact of economic status on the self-assessment of life satisfaction among the elderly, with economic burdens inversely affecting life satisfaction (32). Income adequacy, serving as an indicator of economic well-being, is a vital component in the subjective assessment of an individual’s overall well-being or quality of life (33). Consequently, enhancing the elderly’s self-assessment of life satisfaction necessitates the continual improvement of their economic living conditions, as economic security serves as the fundamental basis for life, safeguarding daily living standards and elevating overall quality of life.

#### 4.1.2 Mental state and its positive correlation with elderly life satisfaction

A positive correlation exists between mental state and the self-assessment of life satisfaction among the elderly. Diminished depression and anxiety levels among the elderly are associated with higher self-assessed life satisfaction. As individuals transition into old age, physiological decline, weakened social networks, and the loss of close relatives elevate the prevalence of negative psychological traits among the elderly, impacting both perceived health and mortality risk (21, 34, 35). A survey indicates that over 80% of elderly individuals in China experience varying degrees of psychological issues, with approximately 27% facing anxiety and depression, adversely affecting physical and mental health (36). Notably, depressive symptoms within the elderly group are substantial, with path coefficients exceeding 0.7 for mental status, reaching 0.95 in urban elderly populations. Timely attention to and guidance on depressive symptoms among the elderly are essential, preventing the emergence of functional mental disorders that significantly diminish the quality of life (37). Hence, increased focus on the mental state of the elderly and subsequent improvement are imperative to enhance their self-assessment of life satisfaction (38).

#### 4.1.3 Personal status and its negative correlation with elderly life satisfaction

The study reveals that the self-assessment level of life satisfaction among the elderly increases with age. Older individuals in later stages of old age exhibit a greater capacity to adjust attitudes towards life and accept challenges compared to those in earlier stages. This ability to embrace strengths and weaknesses contributes to improved self-assessment of life satisfaction (39). While certain studies propose a negative or non-correlative relationship between age and the self-assessment of life satisfaction among the elderly, further research is warranted based on distinct sample groups (40). Notably, gender differences were observed, with older women exhibiting higher levels of self-assessment of life satisfaction than older men. Discrepancies exist in current research on gender-based differences in the self-assessment of life satisfaction among older adults (22). However, supporting studies posit that women’s proficiency in expressing negative emotions may contribute to higher self-assessment levels in older women than men (41, 42). In terms of education, this study showed a negative linkage of education with the improvement of life satisfaction of the elderly. The possible reason for this observation is that older people with national education have higher demands and standards in terms of their life, which are related to the reality of not being able to meet such standards and demands. However, some scholars have pointed out that the life satisfaction of older adults with higher levels of education should be better than that of older groups with lower levels of education (43, 44). This suggests that there is a complexity in the impact of education on life satisfaction of older adults, and the need for more in-depth study in future by focusing on the combination of more factors (43, 45).

#### 4.1.4 Child care and its positive relationship with elderly life satisfaction

The regular visits of children, communication with the elderly, and financial assistance contribute to an elevated self-assessed level of life satisfaction among the elderly (46). Significantly, children’s visits exert a more considerable impact compared to communication contact, while the influence of financial support is comparatively smaller. This underscores that the enhancement of elderly life satisfaction is primarily derived from children’s care, with visiting surpassing communication contact, and financial support playing a smaller role than psychological care. Research emphasizes the importance of children’s support and care as influential factors in the self-assessment of life satisfaction among the elderly. However, for happiness in later years, support behavior exhibits weaker explanatory power for improving the elderly’s self-assessed quality of life, with children’s attitudes toward the elderly playing a stronger role. The combination of “filial piety” and “filial behavior” emerges as a critical factor in enhancing the self-assessment of life satisfaction among the elderly (47–49).

### 4.2 Regional variances in the impact of depression and economic status

Comprehensive analysis of the structural equation model of self-assessment of life satisfaction for urban, township and rural older adults revealed that there is a difference in the impact of the same influencing factor on the self-assessment of life satisfaction of older adults in different regions. To further validate this difference, we included regional indicators (I4) in Model IV. By observing the influence coefficients and the direction of action of each indicator in Model IV, we found that there were only differences in the intensity of action of each indicator, but not any change in the direction of action. These findings further verified the credibility of the results of Models I-III.

#### 4.2.1 Regional disparities in the influence of depression on elderly life satisfaction

The path coefficients of the observed variable of depression to the latent variable of mental state exhibit a consistent decrease from urban to rural areas, measuring 0.95 and 0.87, respectively. This suggests a diminishing impact of depression on the mental state of the elderly in urban areas compared to towns. These findings align with a comprehensive evaluation study, incorporating thirteen studies and indicating a higher risk of depression among older adults residing in urban areas than in rural areas. Adjusting for covariates further emphasized a significantly elevated risk of depression in urban areas, potentially attributed to higher population density, increased mobility, high-rise and unitized urban dwellings, and decreased human contact. Such factors contribute to decreased familiarity with neighbors, heightened social isolation, and loneliness, subsequently elevating the risk of depression and anxiety (29, 50, 51). Additionally, the accelerated urbanization process, coupled with rural-to-urban migration, and an increasing number of empty-nested elderly in rural areas, may further escalate the risk of depression in rural areas (51).

#### 4.2.2 Regional variances in the impact of economic status on elderly life satisfaction

The influence of economic status on the self-assessment of life satisfaction exhibits distinct patterns between urban and rural areas. In urban settings, the impact of psychological status is more pronounced than that of economic status, while the reverse holds true for township areas. From a demand theory perspective, the relationship between economic status and self-assessment of life satisfaction is nonlinear. When economic resources primarily fulfill residents’ material needs, economic status becomes a significant influencing factor; however, as material needs are met, non-material needs gain importance, attenuating the impact of economic status on self-assessment of life satisfaction (32, 52). Given the higher economic level, superior urban planning, medical conditions, and supporting facilities in urban areas, the influence of economic status on the self-assessment of life satisfaction is relatively weaker among urban elderly residents compared to their counterparts in rural areas.

## 5 Conclusion

Self-assessment of life satisfaction of the elderly is influenced by a variety of different factors. Through the study, we found that economic status, psychological status, and child care are positively related to the self-assessment of life satisfaction of the elderly. Personal situation is negatively related to the self-assessment of life satisfaction of the elderly, showing the older the elderly the higher the self-assessment of life satisfaction, and the self-assessment of life satisfaction of female elderly is higher than that of male elderly. Self-assessment of life satisfaction of the elderly group in urban areas is weaker than that in rural areas due to economic factors, but stronger than that in rural areas due to psychological conditions. Therefore, it is necessary to take targeted measures to improve the life satisfaction of the elderly according to the different characteristics of cities, villages and towns, and to pay more attention to those who have just entered old age and to the male elderly.

## 6 Limitation

In the initial phase of our study, we endeavored to incorporate pertinent indicators, including health level, type of residence, marital status, and social support. However, during the process of integrating these indicators into the structural equations for modeling, challenges arose. The structural equation model either failed to converge or exhibited suboptimal fit. This discrepancy may be attributed to existing correlations between these indicators and other variables within our sample. Further investigation is warranted to delve into the intricacies of these relationships and uncover the underlying reasons for this observed phenomenon.

## Data availability statement

Publicly available datasets were analyzed in this study. This data can be found here: https://opendata.pku.edu.cn/dataverse/CHADS?q=&types=dataverses%3Adatasets&sort=dateSort&order=desc&page=2.

## Author contributions

JY: Formal analysis, Methodology, Writing – original draft. SW: Conceptualization, Writing – review & editing. CL: Data curation, Resources, Writing – original draft. YL: Supervision, Writing – review & editing.
